# Identification of a novel homozygous nonsense mutation in EYS in a Chinese family with autosomal recessive retinitis pigmentosa

**DOI:** 10.1186/1471-2350-11-121

**Published:** 2010-08-10

**Authors:** Yukan Huang, Jing Zhang, Chang Li, Guohua Yang, Mugen Liu, Qing K Wang, Zhaohui Tang

**Affiliations:** 1 Key Laboratory of Molecular Biophysics of Ministry of Education, College of Life Science and Technology, Center for Human Genome Research, Huazhong University of Science and Technology, Wuhan, Hubei, PR China; 2 Union Hospital, Huazhong University of Science and Technology, Wuhan, Hubei, PR China; 3 Department of Genetics, School of Basic Medical Science, Wuhan University, Wuhan, Hubei, PR China

## Abstract

**Background:**

Retinitis pigmentosa is the most important hereditary retinal degenerative disease, which has a high degree of clinical and genetic heterogeneity. More than half of all cases of retinitis pigmentosa are autosomal recessive (arRP), but the gene(s) causing arRP in most families has yet to be identified. The purpose of this study is to identify the genetic basis of severe arRP in a consanguineous Chinese family.

**Methods:**

Linkage and haplotype analyses were used to define the chromosomal location of the pathogenic gene in the Chinese arRP family. Direct DNA sequence analysis of the entire coding region and exon-intron boundaries of *EYS *was used to determine the disease-causing mutation, and to demonstrate that the mutation co-segregates with the disease in the family.

**Results:**

A single nucleotide substitution of G to T at nucleotide 5506 of EYS was identified in the Chinese arRP family. This change caused a substitution of a glutamic acid residue at codon 1,836 by a stop codon TAA (p.E1836X), and resulted in a premature truncated EYS protein with 1,835 amino acids. Three affected siblings in the family were homozygous for the p.E1836X mutation, while the other unaffected family members carried one mutant allele and one normal EYS allele. The nonsense mutation p.E1836X was not detected in 200 unrelated normal controls.

**Conclusions:**

The *EYS *gene is a recently identified disease-causing gene for retinitis pigmentosa, and encodes the orthologue of *Drosophila *spacemaker. To date, there are only eight mutations in *EYS *that have been identified to cause arRP. Here we report one novel homozygous nonsense mutation of *EYS *in a consanguineous Chinese arRP family. Our study represents the first independent confirmation that mutations in *EYS *cause arRP. Additionally, this is the first *EYS *mutation identified in the Chinese population.

## Background

Retinitis pigmentosa (RP; OMIM 268000) is characterized by the constriction of the visual fields, night blindness, changes of the fundi including 'bone corpuscle' lumps of pigment, and the loss of central vision. The worldwide prevalence of RP is about one in 4,000 [1]. RP is inherited most frequently (50-60% of cases) as an autosomal recessive trait, followed by autosomal dominant (30-40%) and then X-linked (5-15%) [1-6]. It is the most common hereditary retinal dystrophy causing irreversible blindness [1-3]. About 30 genes and loci have been implicated in isolated cases of arRP to date [RetNet: http://www.sph.uth.tmc.edu/Retnet/disease.htm]. Mutations in *ABCA4*, *CDHR1*, *CERKL*, *CNGA1*, *CNGB1*, *CRB1*, *IDH3B*, *LRAT*, *MERTK*, *NR2E3*, *NRL*, *PDE6A*, *PDE6B*, *PRCD*, *PROM1*, *RBP3*, *RGR*, *RHO*, *RLBP1*, *RP1*, *RPE65*, *SAG*, *SPATA7*, *TULP1*, *USH2A *and most recently *EYS *have been identified to be the cause for arRP [7,8]. However, the gene(s) causing sporadic arRP in most families has yet to be discovered. The identification of genotypes and phenotypes and the physiological roles of novel RP genes in the retina should provide valuable information for the diagnosis and classification of retinal degeneration. Consequently, genetic studies will result in the scientific basis for the prevention and treatment of RP.

RP25, a genetic locus for arRP, was mapped to a ~ 16 cM region on chromosome 6p12.1-q15 in four Spanish families by Ruiz et al. in 1998 [9]. Then, the pathogenic genes for several arRP families with various ancestral origins, including one Pakistani family and three Chinese families, were also been mapped to this locus [10-12]. By using the 10K genechip array, Barragán I et al. refined the disease interval from the original 16 cM to only a 2.67 cM region between D6S257 and D6S1557 [13]. Most recently, the disease-causing gene at this locus has been identified as the *EYS *gene. EYS is predicted to be a 3,165 amino acid multi-domain protein, which contains at least 28 epidermal growth factor (EGF)-like domains in its N-terminus, and C-terminal laminin A G-like domains. It is an evolutionarily conserved protein from Drosophila to humans. Finally, eight *EYS *mutations were detected in eight arRP families from Spanish and Dutch origins at almost the same time in 2008 [7,8].

In this study, we characterized a consanguineous Chinese family with a severe form of arRP. After linkage analysis with known arRP causative genes and loci, we excluded all other arRP loci except for the RP25 locus. Further direct DNA sequence analysis revealed a homozygous mutation in the *EYS*. Our data confirmed the conclusion from the two original reports that mutations in *EYS *can cause arRP, and expanded the mutation spectrum of *EYS *to the Chinese population.

## Methods

The study subjects from a consanguineous Chinese arRP family were recruited from Hubei Province, P. R. China. Informed consent was obtained from the participants in accordance with the study protocols approved by the Ethics Committee of Huazhong University of Science and Technology. Diagnosis of RP was carried out by clinical and ophthalmological examinations. Peripheral blood was collected from the family members and 200 normal Chinese Han controls. Total human genomic DNA was isolated with the DNA Isolation Kit for Mammalian Blood (Roche Diagnostic Co., Indianapolis, IN).

Linkage and haplotype analyses were applied to test the linkage of the family to 22 arRP loci, including *ABCA4, CERKL, CNGA1, CNGB1, CRB1, EYS, I8DH3B, LRAT, MERTK, NR2E3, NRL, PDE6A, PDE6B, PRCD, PROM1, RGR, RHO, RLBP1, RPE65, SAG, TULP1*, and *USH2A*. The microsatellite markers flanking these loci were selected from Linkage Mapping Set MD-10 (Applied Biosystems, Inc., Foster City, CA), and genotyped using an ABI 3100 Genetic Analyzer. Genotypes were analyzed by the GeneMapper 2.5 Software program (Applied Biosystems, Inc., Foster City, CA). PCR and genotyping were performed as previously described [14]. When the pathogenic gene was mapped to chromosome 6q12 that harbors the *EYS *gene, additional SNPs (rs4710437, rs1057530, rs4710457, rs66462731, rs10944813, rs4710292) were employed for the linkage analysis to get unambiguous linked haplotype. The *EYS *was then subjected to mutational analysis. All coding exons and splice sites of *EYS *were amplified by PCR and sequenced. The intronic primers and corresponding PCR conditions were as described in the paper of Abd El-Aziz et al. [7].

## Results

**Figure 1:**
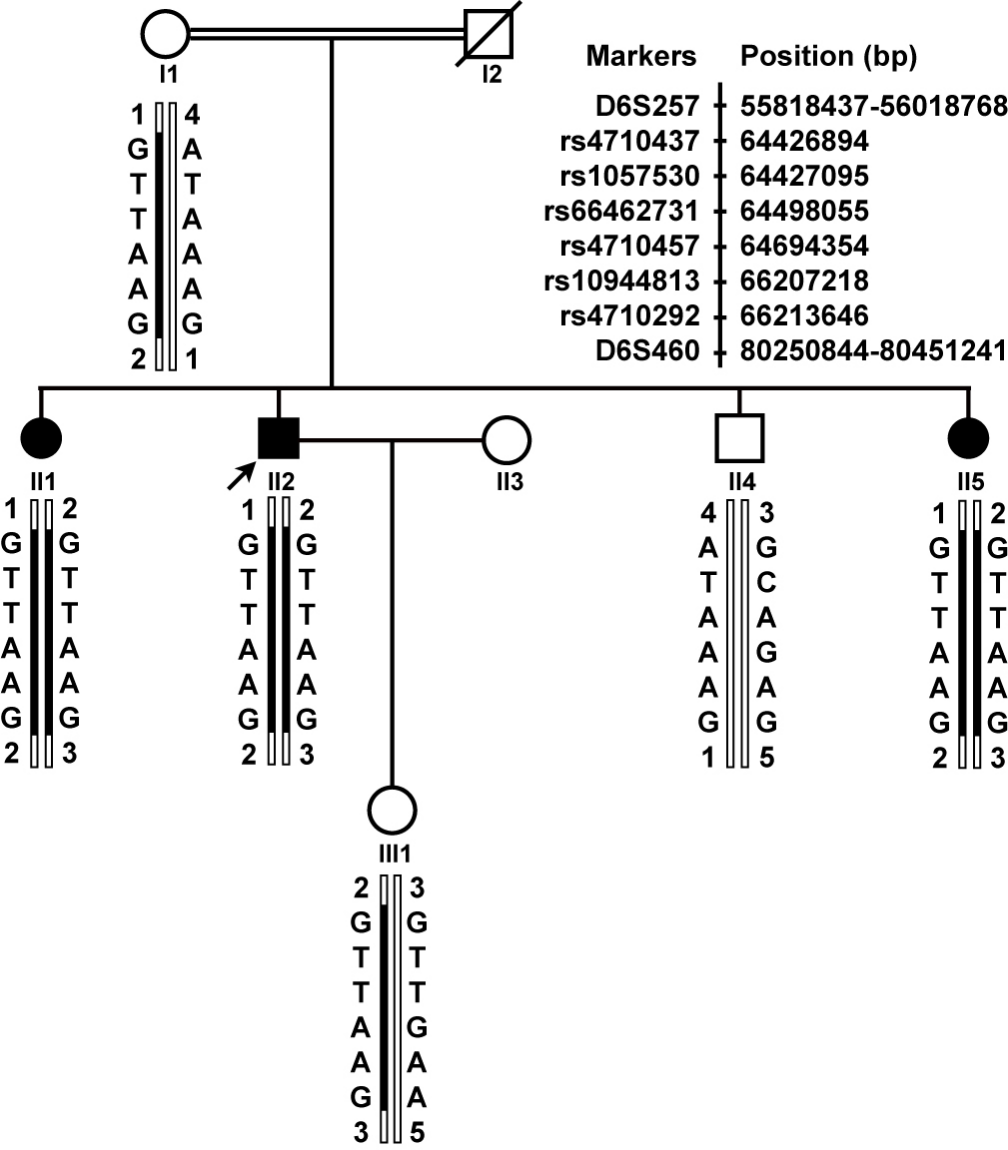
**Pedigree structure and haplotype analysis at the RP25 locus**. Blackened bars indicate the disease haplotype. Filled squares or filled circles represent male or female individuals affected with RP, respectively. Arrow points to the proband (II2). All patients in the arRP family carry the homozygous haplotype between single nucleotide polymorphisms rs4710292 and rs4710437. The genomic positions of two markers are from Human (*Homo sapiens*) Genome Browser Gateway, the GRCh37 build version, and six SNPs are from NCBI B37.1 assembly.

The RP in the consanguineous Chinese family is inherited in an autosomal recessive mode (figure 1). Parents I1 and I2 are third cousins. The proband II2 and his two sisters had deteriorating vision, while the parents and young brother II4 did not show any RP features. The family history was negative for deteriorating vision or night blindness.

**Figure 2:**
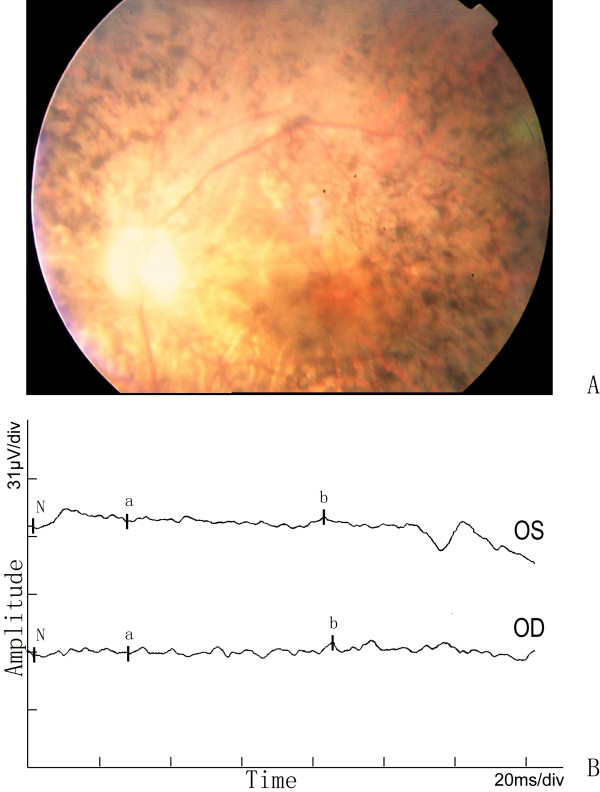
**Fundus photographs and ERG of the proband in the Chinese arRP family**. The features of waxy-pale disc, arteriolar attenuation, and bone-spicule pigment deposit in the mid-peripheral retina are shown in A. The full-field ERG in the proband in B, either a-wave or b-wave has obvious reduced amplitude and prolonged response time in both eyes.

The proband exhibited night blindness at the age of 15 years, and progressively lost his visual acuity to 20/400 at approximately the age of 35-40 years. Funduscopic examinations revealed obvious attenuation of the retinal arteries, waxen color of discs, and bone-spicule pigmentation in the peripheral-mid retina (figure 2A). Electrophysiological examination showed an extinguished or very low amplitude ERG in both eyes (figure 2B).

The other two affected individuals also had the similar symptoms as the proband. Night blindness appeared late in the second decade of life, low visual acuity between 20/400 and hand movement, and a flat ERG confirmed the diagnosis and severity of the RP disease. No additional symptoms or ocular defects were observed in the affected individuals of the family except for patient II5, who was affected with cataract in both right and left eyes.

After linkage analysis for known arRP causative genes and loci, there was only the RP25 locus that showed a definite linkage with the disease in the family. Homozygosity mapping with haplotype analysis revealed that all three affected siblings (II1, II2, II5) inherited the affected alleles separately from their carrier parents, whereas their unaffected brother II4 inherited two normal chromosomes, and the proband's daughter III1 only one affected allele (Figure 1). These results suggest that *EYS *may be the pathogenic gene in this family.

**Figure 3:**
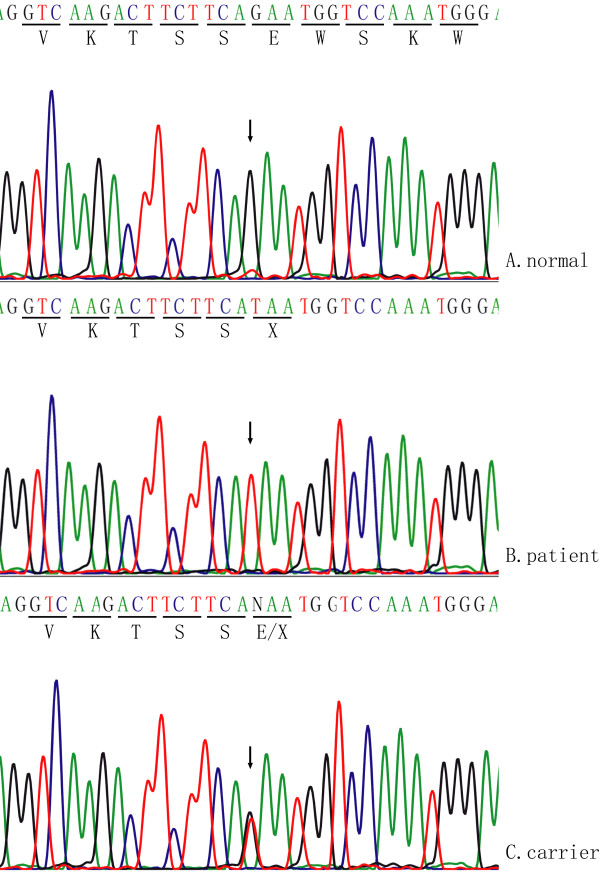
**Identification of the homozygous mutation in the consanguineous Chinese arRP family**. A and B show the exon 26 of *EYS *sequences from a normal individual and an affected individual with p.E1836X mutation, respectively. The sequence for individual III1, who is heterozygous for the mutation, is shown in C.

Direct DNA sequence analysis for the entire coding region of *EYS *(Genbank accession No: FM209056) found a single nucleotide transversion of G to T at nucleotide 5506 of *EYS*, and it resulted in a substitution of a glutamic acid residue at codon 1836 of EYS by a stop codon TAA (p.E1836X) (Figure 3). The nonsense p.E1836X mutation generates a truncated EYS protein with only 1835 amino acid residues. Three affected siblings are homozygous for the p.E1836X mutation, whereas their mother, and III1, the daughter of proband, carry only one affected allele. The p.E1836X mutation was not detected in 200 unrelated normal controls.

## Discussion

A novel *EYS *nonsense mutation p.E1836X in a consanguineous Chinese arRP family was identified, which is the first *EYS *mutation discovered in the Chinese population. The p.E1836X mutation is predicted to generate an abnormal EYS protein with only 1,835 of 3,165 amino acid residues. EYS is a multi-domain protein containing 28 epidermal growth factor (EGF)-like domains, and 5 LamG domains at the C-terminus. All 5 LamG domains and 7 EGF-like domains in the C-terminus of EYS were truncated in the arRP family, which may significantly disrupt the normal physiological function of EYS in the retina.

The *EYS *gene has recently been identified as the disease-causing gene for retinitis pigmentosa, and encodes an orthologue of *Drosophila *spacemaker. So far, only eight mutations have been reported in arRP families by two groups in 2008. El-Aziz et al identified six mutations, including four homozygous mutations p.D904QfsX17, p.S754AfsX6, p.T657TfsX5, p.W2640X and two compound heterozygous mutations p.E1953X and p.R589RfsX5 [7]. Collin et al. reported a p.Y3156X in two unrelated Dutch families and a p.P2238PfsX16 in an isolated RP patient from Dutch origin [8]. Interestingly, all of these reported mutations, including our finding, resulted in a truncated EYS protein. This suggests that the C-terminus of EYS is essential for its function in the retina, or these EYS mutations (nonsense mutations, deletions) may cause RP by degrading the *EYS *mRNA through a nonsense-mediated decay mechanism.

RP 25 could be a major locus for arRP because 10-20% of Spanish typical arRP families were found to have mutations in *EYS *[9,13]. Consequently, it is interesting to study the relationship between the clinical features of RP and different *EYS *mutations. Until recently, mutations of *EYS *have been detected in eight families [7,8]. Three Chinese families were linked to the RP25 locus, but no mutations were identified in the *EYS *gene [12]. Similarly, in one Pakistani arRP family, RP was suggested to be caused by an *EYS *mutation, too [10]. All the patients in these families displayed typical characteristic RP symptoms, including night blindness as the initial symptom, retinal bone-spicule pigmentations, attenuated retinal vessels constriction of visual fields, and absent or flat ERG. However, the age of onset displayed interfamilial differences, ranging from the early second decade to the late forth decade. It is notable that two patients (described by Collin et al.) from two different families with mutation p.Y3156X had combined RP and cataract [8]. One patient II5 in this study was also affected with cataract in both right and left eyes. Further work on deciphering if EYS plays an important role in the cataractogenesis in addition to retinal dystrophy may be useful to understand the function of EYS.

*EYS *contains 44 exons covering 2.0 Mb, and it is considered to be the largest gene identified and expressed in the human eye as well as the fifth largest overall in the human genome. *EYS *is highly expressed in the retina, but its biological function remains unknown. In *Drosophila*, the EYS homological protein *spam*, and its interactor protein *prom*, plays a critical role in retina morphogenesis. *Drosophila *mutant lines for *spam *and *prom *can result in a failure of inter-rhabdomeral-space separation [15]. Mutations in human *PROM1 *were associated with arRP, macular degeneration, and cone-rod dystrophy [16-19]. *EYS *mutations causing arRP and the data from *Drosophila *strongly suggest that EYS may have an important function in the photoreceptor morphogenesis. Further investigations of the biochemical function of EYS in retinas will lead to a significant understanding of the pathological mechanism(s) of retina degeneration.

## Conclusions

A novel homozygous nonsense mutation in EYS, p.E1836X, was responsible for using arRP in the consanguineous Chinese family. This is the first *EYS *mutation found in the Chinese population. This research confirms that mutations of *EYS *can cause arRP. It has also expanded the spectrum of *EYS *mutations in arRP.

### Note Added in Proof

Very recently, three groups of investigators described the identification of *EYS *novel mutations in patients with retinal dystrophy. Abd El-Aziz et al. [20] identified novel EYS mutations in arRP patients from British and Chinese origins. Bandah-Rozenfeld et al. [21] reported EYS mutations in Israeli and Palestinian families with autosomal recessive retinitis pigmentosa (arRP). Audo I et al. [22] found *EYS *mutations in French patients with rod-cone dystrophies. These studies supported that *EYS *is critical for retinal function and confirmed that *EYS *is a major causative gene for retinal dystrophies in populations of various ethnicities.

## Competing interests

The authors declare that they have no competing interests.

## Authors' contributions

YH recruited the members of arRP pedigree, performed clinical studies for whole family members, and relationship studies between genotype and phenotype. JZ carried out the extraction of genomic DNA and genotyping assay. CL was responsible for the extraction of genomic DNA and sequencing for mutation detection. GY was involved in linkage analysis and the discussion of the manuscript. ML participated in the original conception of this study, and contributed to the revision of the draft. QKW conceived and designed the experiments and helped the critical revision of the manuscript. ZT designed the experiments, analyzed the data, interpreted the results, and drafted the manuscript. All authors read and approved the final manuscript.

## Pre-publication history

The pre-publication history for this paper can be accessed here:

http://www.biomedcentral.com/1471-2350/11/121/prepub
